# Settlement of Soil Reinforced with Vertical Fiberglass Micro-Piles

**DOI:** 10.3390/ma15144744

**Published:** 2022-07-07

**Authors:** Mohanad Muayad Sabri Sabri, Nikolai Ivanovich Vatin, Andrey Budimirovich Ponomarev, Renat Rustamovich Nurmukhametov, Ivan Igorevich Kostyukov

**Affiliations:** 1 Peter the Great St. Petersburg Polytechnic University, 195251 Saint Petersburg, Russia; vatin_ni@spbstu.ru (N.I.V.); andreypab@mail.ru (A.B.P.); 2 Department of Construction and Urban Planning, Saint Petersburg State Forest Technical University, 190999 Saint Petersburg, Russia; spb.kostyukov@mail.ru

**Keywords:** fiberglass, composite reinforcement, soil reinforcement, FRP pile, screw micro-pile, fiber-reinforced composite

## Abstract

This article is dedicated to investigating the properties of soil after its reinforcement with fiberglass elements through large-scale laboratory plate-load tests of various samples that varied in the numbers and lengths of the reinforcing elements. The investigation of the vertical elements considered the diameter increase at the bottom toe by using widening washers. The results were compared relative to each other and to the theoretical calculation results. The theoretical calculations for the settlements were undertaken based on the authors’ proposed method. The method considers the number, shape, area and material of the strengthening elements using a pre-proposed reinforcement area factor µ. This pre-established factor was calculated with reference to the elements’ geometry—the diameter of the vertical elements and the bottom’s washer diameter—which determined the reinforcement area. A comparison between the reinforced and reference soft sandy soil samples indicated a 25% increase in the deformation modulus after the reinforcement process at a pressure of 25 kPa. Samples with µ ranging from 1.20 to 1.43 were 55–65% stiffer than samples with µ equal to 0.69 at a pressure of 100 kPa. The comparative analysis of the calculated results and the actual laboratory PLT test results was adequate for use for further development.

## 1. Introduction

The reduction of foundation settlements is often required in various practical geotechnical tasks due to soft soil conditions, reconstruction of existing constructions and infrastructures and other reasons. Deepening the foundation’s bottom level is the most often used approach nowadays. However, this approach has several disadvantages and requires the attention of a highly qualified team. Replacement of the soil is not feasible in existing constructions due to various technical and economic reasons. Hence, the vertical reinforcement of soil is one of the techniques used to improve ground strength and reduce foundation settlements. The term “reinforcement” refers to the strengthening of soil through penetration of additional elements with higher strength properties and different shapes and materials into the soil mass.

Nuzhdin et al. modeled vertical soil reinforcement with cement grouting in laboratory trays by using gravel workings as a hard inclusion [1]. Nascimento et al. [2] described an experiment involving the application of sand columns within soft clays, which, besides reinforcement, played a role in drainage.

The search for materials to use for the strengthening of soil is an appropriate direction of research from a sustainable engineering point of view since recycled materials can be applied. Thus, Shenkman and Ponomaryov reinforced soil with stone columns encased in a fiberglass geotextile [3]. A method for soil reinforcement using cased piles was studied by Fattah and Majeed [4].

Yeung et al. compared vertical reinforcement using galvanized steel bars with glass and carbon fiber-reinforced polymers (GFRPs/CFRPs) installed in soil and pressure grouted with cement [5]. The authors considered fiber-reinforced polymer (FRP) pipes as injectors and, further, as soil reinforcements. The system is used in Korea and Hong Kong to increase the shear strength of weak soil slopes; to reinforce cut slopes, tunnels, and excavations; to construct seepage cutoffs; and to stabilize fault zones or other discontinuities in rock masses. Sabri et al. studied soil reinforcement methods involving the injection of polyurethane resin [6,7,8,9].

The underpinnings of natural foundations have been studied by Waruwu et al. [10]. Reinforcement was modeled using vertical elements and a horizontal bamboo grid.

Recent research has considered the application of reinforcement elements involving a fiberglass pultruded pipe with a screw-shaped toe made of a cast iron screw [11]. This design for screw-type vertical elements offers many advantages, such as easy installation and improved interaction between the reinforcing element and the surrounding soil. These samples make good use of the advantages of fiberglass. Various advantages are gained by using the FRP soil reinforcement method rather than steel reinforcement techniques, and Zyka and Mohajerani [12] list the following benefits of fiberglass materials and structures:

- The material is corrosion-resistant and, hence, it reduces maintenance expenses. FRP structures do not require primer coating and further painting, and this especially is essential for the comparison with steel substructures. Hence, maintenance is simple, as described by Pando et al. [13].
- Fiberglass has high strength (R_bend_ = 690 MPa) that can be compared to the tensile strength of steel.
- Fiberglass has three to four times lower weight (1600 kg/m^3^) compared to the weight of steel. Thus, Boyarintsev noted an increase in constructability for areas of complicated access; for example, areas where helicopters are used for transportation, such as the Arctic zone [14].
- Fiberglass is a low-thermal-conductivity material (60–80 times lower than steel).
- Fiberglass does not allow electric contact and can be used for lightweight, thin-walled, steel structure frames. A dielectric basement increases the lifespan of these frames due to the absence of corrosion caused by electric contact with the ground.
- The slow obsolescence and long lifecycle of the composite structures provide a positive impact on the environment and bring consumers closer to sustainable engineering.
- Mohajerani and Zyka [12] studied the lifecycle of the SeaPile fiberglass piles produced by Bedford Technology. In comparison to wooden piles, it was proved that fiberglass piles become cheaper after six years in service, considering the maintenance cost.

Tokhi et al. [15] and Sharma et al. [16] have tested similar screw-type structures for soil nails and confirmed the benefits of their use. From their test results, it is observable that the failure of the screw nail satisfies the Mohr–Coulomb failure condition. Nailing is considered a parallel approach to soil reinforcement that is used for the improvement of soil strength parameters, as stated by Franzén [17]. Nailing is mainly applied for stabilization of inclined and vertical surfaces and slopes. Stephens et al. [18] have studied steel spiral nails transformed from bended- into helical-shape steel profiles.

The wide application of the investigated method is still restricted for several reasons, including the absence of an adequate calculation method for the soil reinforced by fiberglass. One of the points investigated by Zhang et al. [19] and Sidhardhan et al. [20] is the interaction between the soil and the reinforcement. Yin et al. have proposed a simplified analytical method for calculating the maximum shear stress of the nail–soil interface [21] and tested it on the soil–cement grout interface shear strength in soil nailing using a direct shear box testing method [22].

The shear strength of FRP materials needs careful attention and depends on the wire allocation during forming glass roving for the produced structure. Hence, additional shear roving increases the shear strength but, consequently, increases the cost. Zhu et al. studied the field pullout performance of GFRP soil nails with a geometry including wideners [23].

Papadopoulou et al. [24] applied FE analysis for the assessment of helical micro-piles. The geometry of the assessed micro-piles was similar to that described for the reinforcing elements within the current paper. Pando et al. [13] and Valez et al. [25] have tested and renovated the structures of composite piles. Valez et al., as well as fiberglass structures, also tested carbon fiber piles in stabilized and non-stabilized soft soils. The results demonstrated higher adhesion and bearing strength compared to steel piles. Guades et al. [26] tested an epoxy coating to resolve the issue of delamination. The epoxy coating was injected between the shell and core, improving the cohesion and dynamic load transfer for the driven piles. Additional investigations are required to decrease the delamination influence on piles’ bearing strength, especially for lateral and torsional forces.

Popov evaluated the layer-by-layer calculation method for soil settlement summation considering the stiffness of the inserted vertical elements [27]. According to Popov, additional tests are required to confirm and further develop the method.

Lv et al. performed comparative analysis for circular-shaped vertical reinforcement and X-shaped cast in situ piles [28]. Several investigations of horizontal reinforcement behavior were undertaken by Bartolomey [29] and Naggar [30]. Zhou studied the horizontal reinforcement of embankments by creating analytical models [31]. Abu-Farsakh et al. proved the benefits of horizontal reinforcement of soft soil with laboratory tests [32]. Chen et al. analyzed the method of reinforcement for weak soils using a geogrid and geotextile composite [33]. Liu proposed an analytical method for the estimation of the loads applied to vertical wrapped-face reinforced soil walls that was initially validated by numerical analysis and finally confirmed with full-scale results. The method assumes the compatibility of soil and reinforcement deformations and is based on the hyperbolic stress–strain relationship of soil and Rowe’s stress dilatancy equation [34].

The materials investigated within scientific papers do not have enough experimental and practical validation. Existing soil reinforcement studies are insufficient to understand the investigated composite structures and their behavior within soft soils. Luchkin et al. [35] noted that methods applied for settlement calculation had low correlations with test results (0.86–0.94). Combining a calculation model that would correlate with test results at a level above 0.95 with algorithms modeled using modern software will bring the studied method closer to practical application.

Additional verification is required through the performance of laboratory and site tests to develop a theoretically and practically proved method of soil reinforcement using vertical elements.

This study aimed to investigate soil properties after its reinforcement with fiberglass elements differentiated by length and cross-section area using large-scale laboratory plate load tests. The results are compared relative to each other and with the pre-determined theoretical calculation results.

This study’s objectives are listed below:

- To determine the load–settlement relationship for soil reinforced by FRP elements.
- To compare test results with the authors’ vertical settlement calculation approach, which was previously formulated and adjusted during the research lifecycle. The latest equations for the FRP reinforcement factor to be applied in sandy soils are described below.
- To review the complex settlement model for soil and reinforcing elements.
- To assess and check, at an early stage, the possibility of further use for the complex settlement model.
- To assess the influence of the increase in the cross-sectional area at the bottom of each reinforcing element on the properties of the FRP-reinforced soil composite.

## 2. Materials and Methods

### 2.1. The Reinforcement Elements and Loading Method

Soil plate load tests were performed to investigate the load–settlement curves of the soils reinforced with FRP. Tests were performed with laboratory buckets that were filled with soft, uncompacted sand.

**Figure 1:**
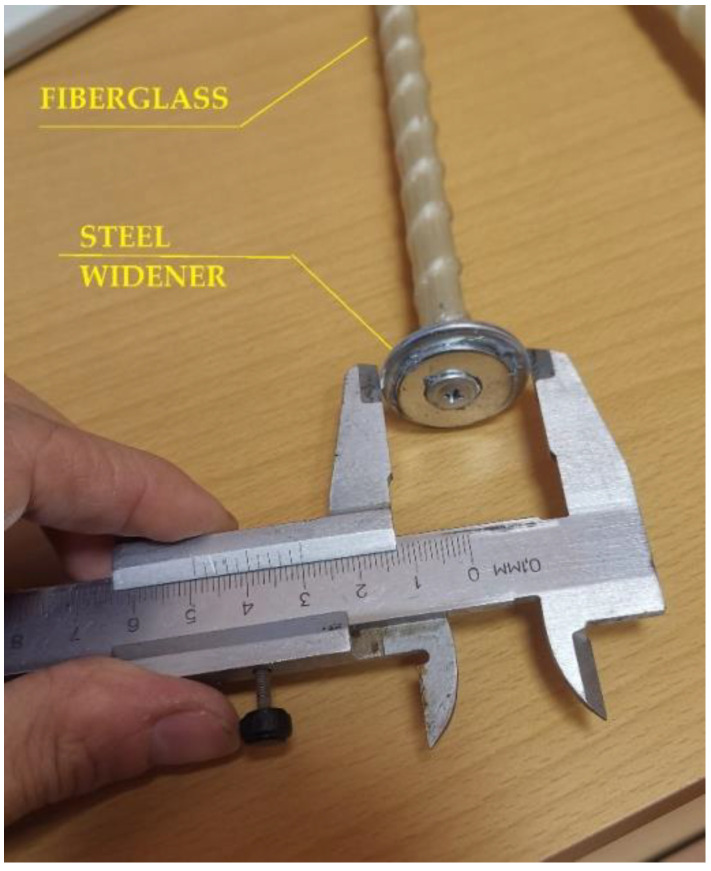
Fiberglass reinforcing elements.

**Figure 2:**
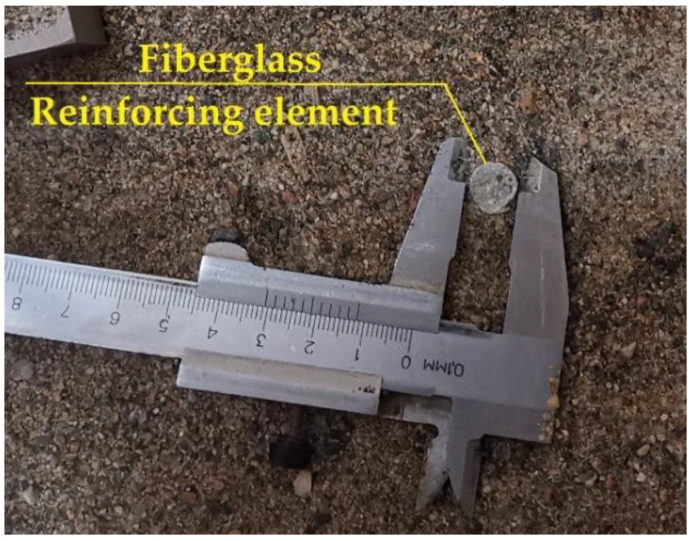
Location of vertical element lowered into the soil.

Samples were reinforced using FRP bars similarly to the reinforcement in steel-reinforced concrete. The diameter of the FRP bars was 12 mm. Twenty-five centimeter lengths were chosen for the reinforcing elements o according to the depth of the testing buckets. Two extra samples were produced, with the length of each increased to 40 cm to perform an elementary check of the optimum length of the reinforcing elements and the influence of length variations on the composite’s properties. Reinforcing elements were fabricated with an increased diameter of 30 mm for the bottom toe. Figure 1 and Figure 2 show the fiberglass reinforcing element used in the experiments.

The fiberglass bars were fabricated with shear-bent rovings to increase the shear connection between the soil and the vertical reinforcing elements. The width increase at the bottom toe was intended to provide better load distribution within the soil-reinforcing composite element.

**Figure 3:**
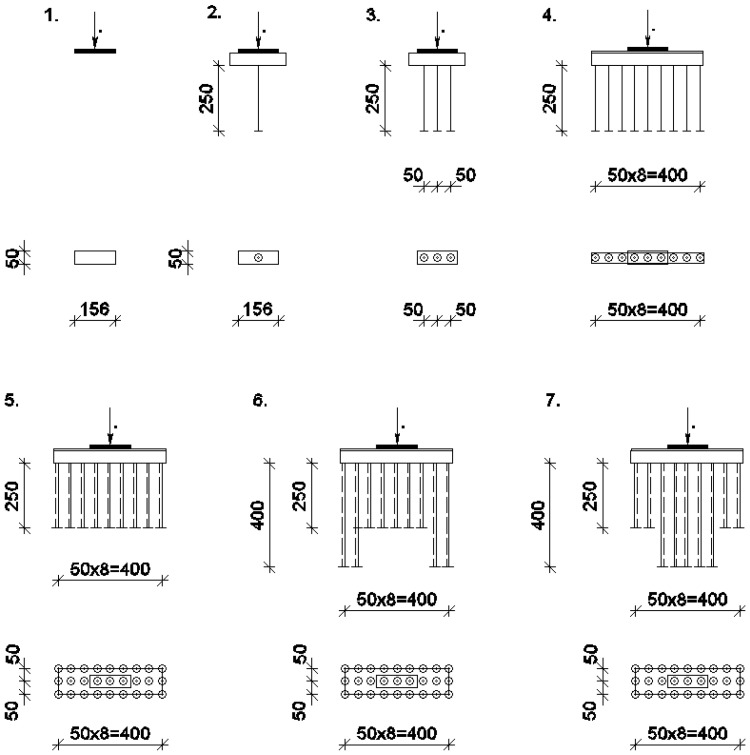
Seven options for the vertical element allocation of the FRP, as tested in the experiments. Dimensions are in mm.

Figure 3 shows the allocation of the vertical elements. Seven different schemes for the reinforcing element distribution samples were developed and tested, as follows:

- Without reinforcing elements;
- One, three, or nine vertical elements located on one line;
- Twenty-seven FRP elements located on three lines;
- Two samples with increased 40 cm length sample allocations.

Each scheme was tested using large-scale laboratory PLTs no less than three times to ensure the accuracy of the obtained results.

Table 1 describes the reinforcement factor for each tested sample. Reinforcement factors were calculated as per Equation (3).

The loading process was carried out using an automated loading machine managed by the software installed on a local computer. The computer was connected to the datalogger by an RS485 cable, and settlement sensors and loading measurements were connected to the datalogger. 

**Figure 4:**
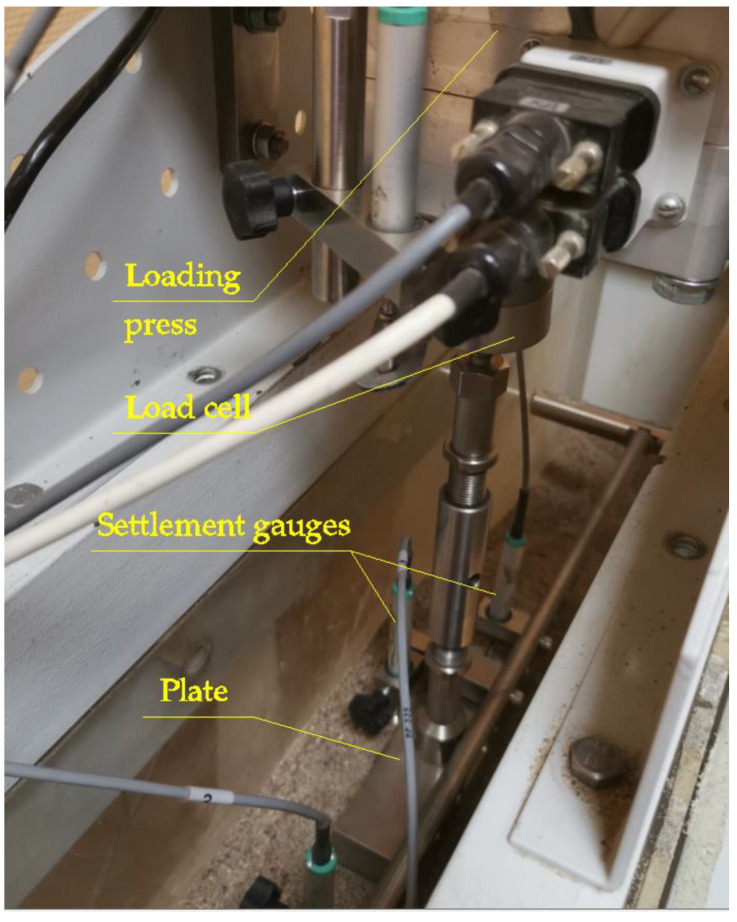
The laboratory plate-load-test measurement system used to perform the experiments.

Figure 4 shows the loading equipment, with a plate and settlement gauges.

Settlements were measured with PZ-12-S-025 spring-type linear gauges (Gefran, Italy). Force measurements were performed using an MNC-2T load cell gauge (C.A.S., Republic of Korea). The buckets, datalogger, and software were produced by GeoTech (Perm, Russia).

Before the loading stages, the parameters of the reference sandy soils were identified in the laboratory using traditional testing methods. Table 2 shows the measured parameters of the investigated uncompacted sand before the reinforcement process.

The samples loading process was performed following the incremental stages of similar tests mentioned by Ponomarev and Sychkina [36]. Loads were increased at each stage by 100 kPa, and a stabilization process was applied between each loading stage.

### 2.2. Background on the Previusly Proposed Theoretical Settlement Calculation Method

As well as the laboratory experiments, the settlement theoretical calculations were determined according to a previously proposed calculation method [37]. The method was developed in the context of the determination of the stress–strain state of water-saturated soils, but it includes the reinforcement factor µ, which is calculated using Equation (3):

(1) $$s_{0}(\mu )=m_{\upsilon }\int_{0}^{h}\sigma (z)dz=\sum_{i=0}^{h}h_{i}m_{\upsilon i}\sigma_{rp}(\mu )=\sum_{i=0}^{h}\frac{h_{i}m_{\upsilon i}N}{A_{rp}(1+\mu \frac{\alpha }{\upsilon })}$$

where:

*σ*(*z*) is the stress on the soil;

$\alpha =\frac{E_{ae}}{E_{rp}}$ is the ratio of the reinforcing element’s elastic modulus to the soil deformation modulus;

*h_i_* is the height of each soil layer;

*A_rp_* is the area of the soil element’s cross-section;

*N* is the vertical force applied to the reinforced soil’s massive;

*v* is the elastic–plastic settlement ratio, calculated as follows:

(2) $$\nu =\frac{\varepsilon_{e}}{\varepsilon_{e}+\varepsilon_{pl}(t,\sigma_{rp}/R_{rp})},$$

*µ* is a reinforcement factor, calculated as follows:

(3) $$\mu =\frac{\gamma_{frn}A_{aef}+\gamma_{Rrn}A_{aeR}}{A_{rp}},$$

*γ_frn_* and *γ_Rrn_* are the factors of the fiberglass reinforcement’s cross-sections and screw areas;

*A_aef_* is the area of the FRP elements’ friction surface;

*A_aeR_* is the area of the reinforcing elements’ screws, imitated by the washer fixed to the bottom toe.

The reference settlement factor $m_{\upsilon i}$ depends on the deformation modulus, *E* which was derived from the lab tests using a modified equation:

(4) $$E_{}=\beta l\frac{\Delta \sigma }{\Delta s^{\prime }},$$

Factor *β* was developed based on Hook’s elastic stress–strain relation boundary conditions. The factor used here depended on a Poisson ratio:

(5) $$\beta =1-\frac{2\upsilon^{2}}{1-\upsilon },$$

**Figure 5:**
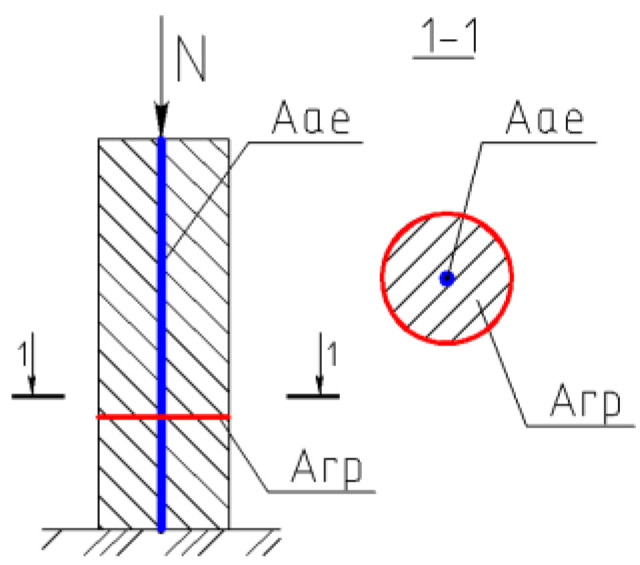
Soil-reinforcing-element model. *A_rp_* is the area of the soil prism’s cross-section and *A_ae_* is the surface area of a single vertical element.

Preliminary studies of soft soil settlement, including theoretical analyses and numerical simulations, were performed considering that the reinforced soil mass is a combined system of soil and reinforcing elements, where the load is distributed between the soil and reinforcing elements. Figure 5 presents the soil-reinforcing-element model. The reinforcing elements are mainly exposed to the load at the first stage due to the higher strength of the reinforcing elements, which act as bearing elements. In the next step, the reinforcing elements penetrated into the soft soil layers, since they were hanged-type elements and not supported by stiffer soil beneath. Hence, some of the soft soils and reinforcing elements were shifted downwards together, acting as a composite material. The analysis approach is similar to the model of soil reinforced by an expandable polyurethane resin developed by Sabri and Shashkin [9] and the reinforcing natural stones described by Korneeva and Sabri [38].

At the reinforcement stage, lowering down the soil between reinforcing elements resulted in greater load due to the application of further load from the plate. Finally, an equilibrium state was achieved. An equilibrium state means that the final tension distribution has been achieved in the reinforced soil through a composite system consisting of soil and vertical reinforcing elements, as described by the following equation:

(6) $$\varepsilon_{ae}=\varepsilon_{rp}=\frac{\sigma_{rp}}{E_{rp}^{\prime }},$$

**Figure 6:**
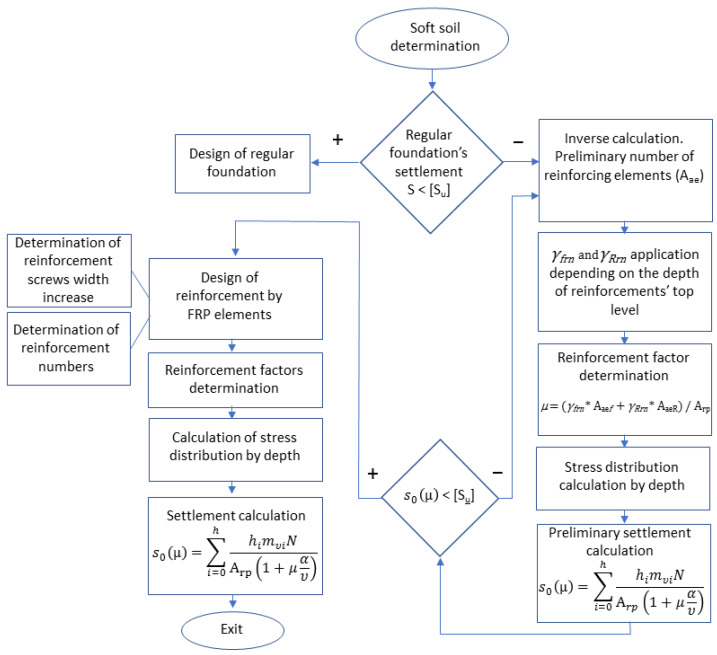
The proposed algorithm for modeling the soil massive reinforcement with FRP vertical elements.

Figure 6 represents the algorithm that was developed based on the proposed calculation formulas.

The proposed settlement calculation method requires additional experimental verification, considering the earlier research of the authors [39], as well as the use of piles to act as vertical reinforcement elements.

## 3. Results and Discussion

### 3.1. Results of the Laboratory Tests

**Figure 7:**
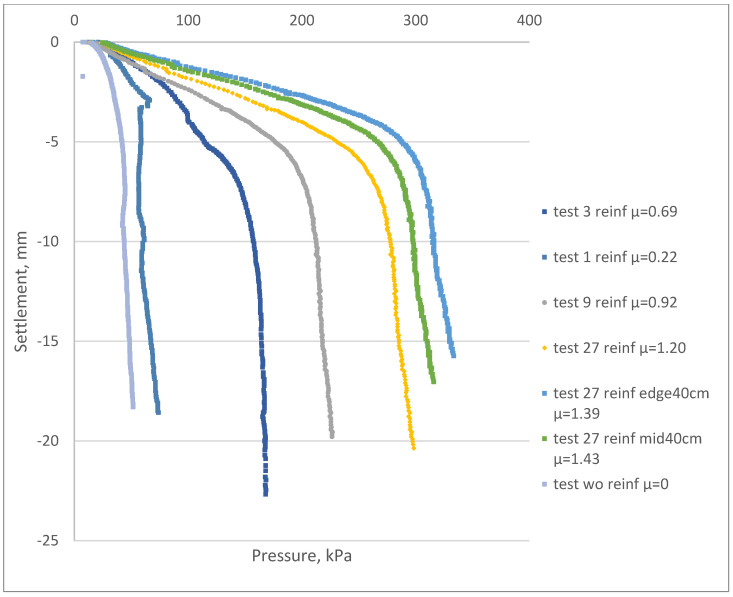
Load−settlement curves for the tested samples before and after the reinforcement process. Reference samples without reinforcement correspond to “test wo reinf µ = 0”. The legend specifies the number of reinforcing elements: 1, 3, 9 or 27 reinf. Elements with 40 cm extended lengths at the edges (sample No 6) or at the middle (sample No 7) are specified as “edge 40 cm” or “mid40 cm” accordingly.

Figure 7 shows the measured load–settlement curves for the reference and reinforced samples.

After a comparative analysis of the results (Figure 7), the following could be concluded:

- Initial test results for the reference and reinforced samples showed an average settlement decrease of 78% at a pressure of 25 kPa after including the reinforcing elements (factor µ equal to 0.22).
- The reinforced soil massive with a reinforcement factor µ ranging from 1.20 to 1.43 was 55–65% stiffer than the samples with a factor µ equal to 0.69 at the pressure of 100 kPa.
- Settlement of the samples with factor µ equal to 1.2 was 41% less than the samples with µ equal to 0.69 at the pressure of 100 kPa.
- It should be noted that the optimum reinforcing element length was equal to the maximum depth of the investigated compacted soil layer under the foundation. A 60% increase in the length of the reinforcing elements (25 to 40 cm) led to a settlement reduction ranging between 21 and 30%.
- The reinforcement factor µ had a direct influence on the overall foundation settlement. Table 2 shows the effect of inclusion of the FRP reinforcement on the increase in the composite deformation modulus.

**Figure 8:**
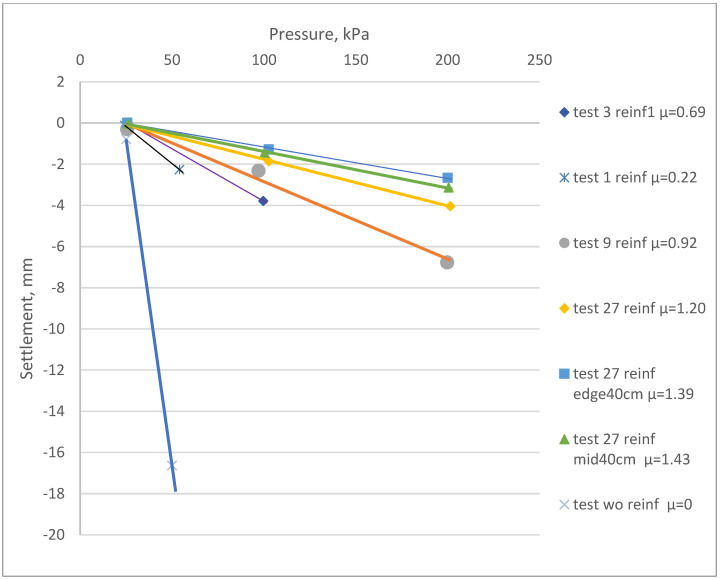
The laboratory PLT elastic deformation for various reinforcement areas before and after the reinforcement. Reference samples without reinforcement correspond to “test wo reinf µ = 0”. The legend specifies the number of reinforcing elements: 1, 3, 9 or 27 reinf. Elements with 40 cm extended lengths at the edges (sample No6) or at the middle (sample No7) are specified as “edge 40 cm” or “mid40 cm” accordingly.

Secant modulus calculation and verification with field plate load tests are still the most reliable approaches. After the compression test results, the primary approach used for settlement method calculations was the calculation of the deformation modulus E at the stress ranges 25–50 kPa, 25–100 kPa and 100–200 kPa (Figure 8). Figure 8 presents the changes in the deformation properties before and after the reinforcement. Table 3 presents a comparison for the variations in the secant deformation modulus.

### 3.2. Comparison with the Results of the Theoretical Settlement Calculation Method

**Figure 9:**
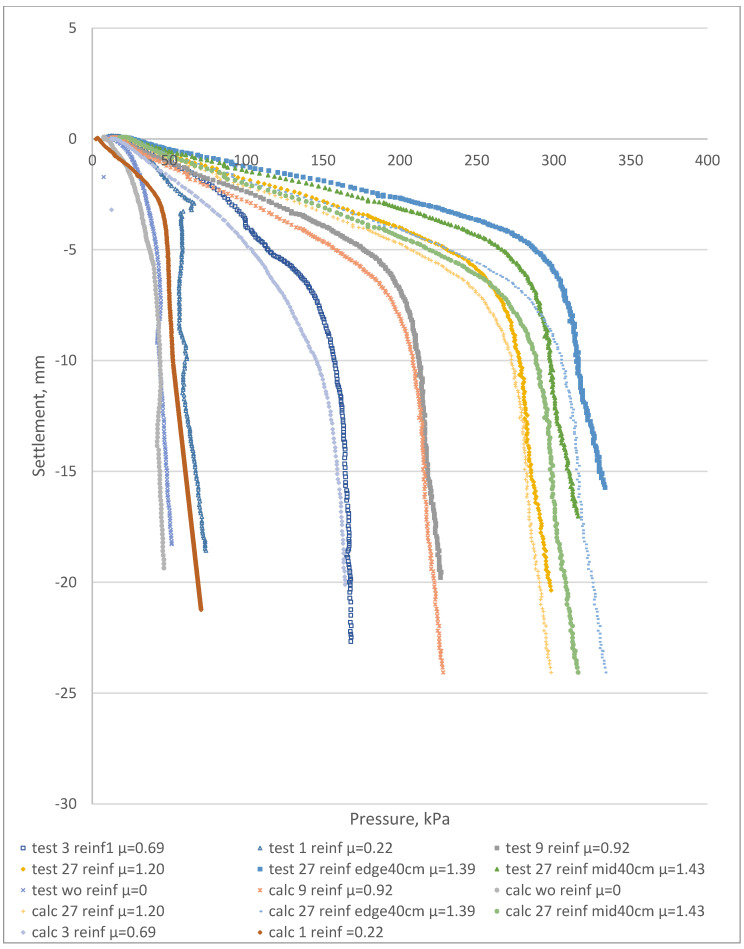
The load–settlement curves for the tested samples compared to the theoretical calculation results. Calculated results are specified by the term “calc”. Test results are specified by the term “test”. Reference samples without reinforcement correspond to “test wo reinf µ = 0”. The legend specifies the number of reinforcing elements: 1, 3, 9 or 27 reinf. Elements with 40 cm extended lengths at the edges (sample No6) or at the middle (sample No7) are specified as “edge 40 cm” or “mid40 cm” accordingly.

The results of the test were used for the comparison with the analytical approach described here. Figure 9 compares the load–settlement curves of the tested samples with the theoretical calculation results from the previously proposed calculation method.

**Figure 10:**
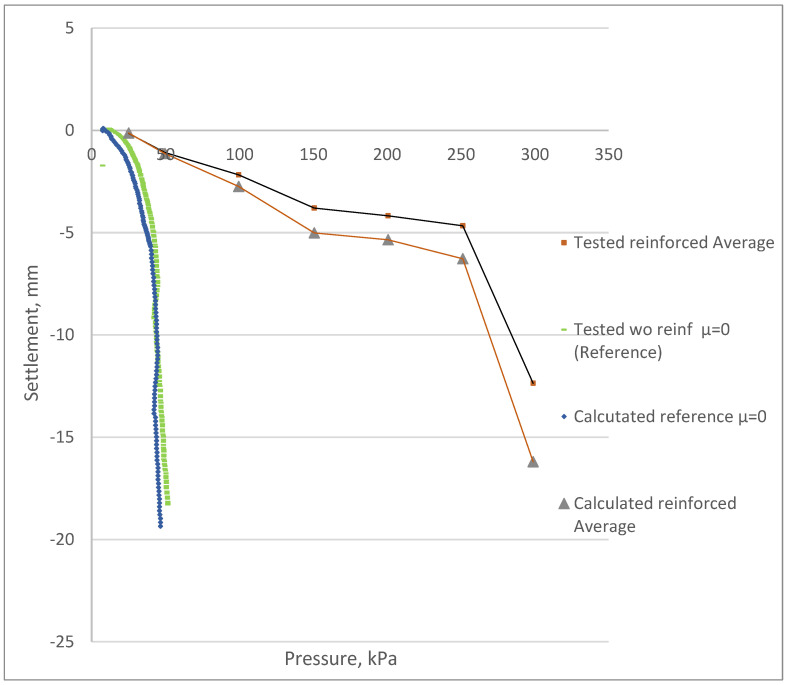
The averaged load–settlement curve for all tested samples compared with the averaged theoretical calculation results before and after the reinforcement process.

A comparative analysis before and after the reinforcement process (Figure 9) showed that the theoretically calculated load–settlement relationships reflected the in situ PLT results with sufficient accuracy. The theoretical calculations were consistent with the obtained laboratory plate load test results within an average range of 5 to 34%. This indicated that the proposed calculation method is reliable for further development and that it confirmed the experimental results, as shown in Figure 10. Although some discrepancies existed between the theoretical calculation and the experiment results, the obtained results represent an attempt to approximate the load–settlement curves according to the elastic behavior of the soil before and after the reinforcement by FRP micro-piles. Large-scale studies performed by the authors of this article have already been published previously [40]. However, further large-scale studies are still required to investigate the change patterns in the FRP-reinforced soil deformation modulus at different soil depths and applied loads.

**Figure 11:**
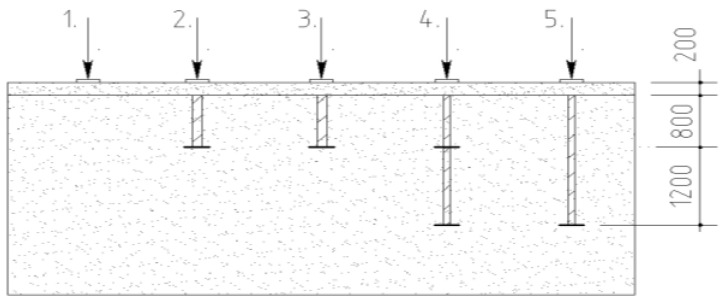
Scheme of the tested samples. 1—reference soil, *µ =* 0; 2—steel screw-type reinforcing element, diameter: 100 mm, µ = 0.23; 3—FRP element, diameter: 100 mm, µ = 0.27; 4—FRP element with two screws, diameter: 75 mm, µ = 0.51; 5—FRP element with single screw, diameter: 75 mm, µ = 0.33.

**Figure 12:**
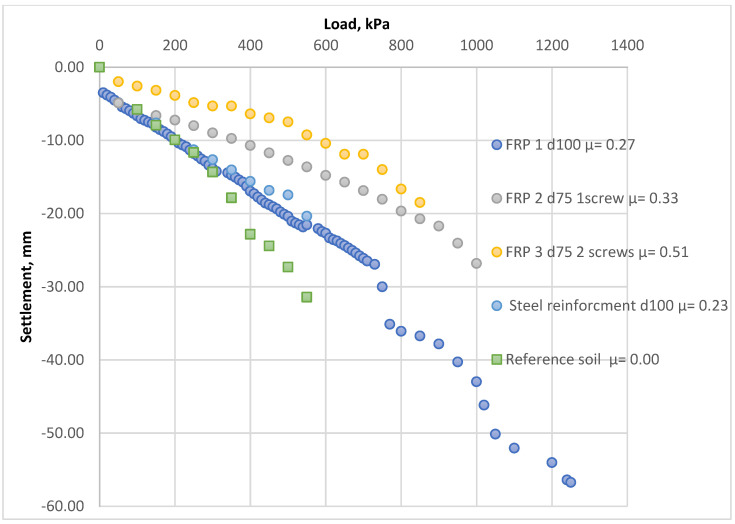
Results of the field tests.

The limited boundary conditions of the bucket influenced the results of the current research. The determination method for the micro-piles’ bearing capacity should be reviewed more carefully, considering the specific calculation details of the micro-piles as reinforcing elements. Hence, full-scale field test results [40] were used to verify the results of the proposed method. The site test included four soil samples reinforced by screw-type elements and one reference sample. The plate load was applied to the samples installed in decompacted sand (Figure 11). The reinforcement factor *µ* was calculated based on Equation (3). Figure 12 presents the load–settlement curves drafted from the results.

**Figure 13:**
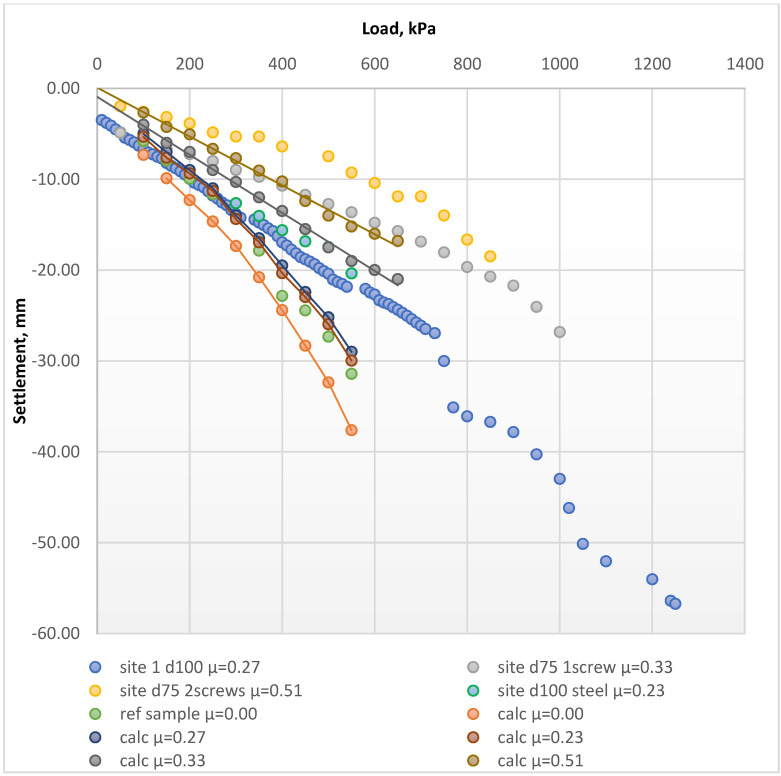
The load–settlement curve for each tested in situ sample compared to the theoretical calculation results. Calculated results are specified with the term “calc”. Site test results are specified with the term “site”. Reference samples without reinforcement correspond to “ref sample µ = 0.00”.

Figure 13 presents the comparison between the settlement values obtained from the natural scale tests and those estimated using analytical solutions. The results of the field load test also verified the proposed analytical method.

Extrapolation of the laboratory results to a full-scale reinforced soil foundation will be performed in the next research stages. Comparisons of the bounded laboratory tests with the site tests and computation model should consider the scale of the reinforcing elements, the boundary conditions and the differences between soil parameters at different sites. It is worth noting that a study of interface behavior between the FRP surface and soil is needed. Further investigations should be performed comparing steel–soil interface behavior. Similar research has already been performed by Frost and Han [41]. FEA modeling of the interaction between soil and FRP structure was performed by Naggar [42].

Investigation into the long-term durability of materials is required. The environmental friendliness of the soil reinforcing elements fabricated in this experiment is apparent. However, a more detailed study of the potential influence on the environment is desirable.

In addition, it must be noted that industrial use of fiberglass structures for soil base strengthening is limited by unconfirmed calculation methods and the subsequent absence of codes.

## 4. Conclusions

Vertical settlements of soil reinforced by vertical FRP elements were studied based on a set of laboratory and site tests. Using the obtained test results, the study can be summarized as follows:

1. A structure for reinforcing elements with fiberglass micro-piles was proposed and confirmed in the laboratory environment.
2. The strength and deformation properties were increased after the inclusion of the FRP reinforcing elements. The average decrease of the settlement was 80% at a pressure equal to 25 kPa after including the reinforcing elements (*µ* equal to 0.22).
3. At a loading pressure of 100 kPa, increasing the reinforcement area’s factor *µ* from 0.69 to 1.2 led to a corresponding decrease in the settlement by 42%.
4. At a loading pressure of 100 kPa, increasing the reinforcement area’s factor *µ* from 0.69 to the range from 1.20 to 1.43 led to a corresponding decrease in the settlement by 55–65%.
5. Comparing the laboratory tests to the theoretically calculated settlement results with the same element geometry based on a previously proposed calculation method demonstrated adequate consistency. Although the deviation in the theoretical and experimental results ranged between 5–34%, the comparison proved that the correct method had been used, and the obtained results can serve for further development.
6. The deformation modulus calculated from compression tests was sometimes far from the actual in situ modulus since sample extraction from the borehole led to tension reduction in the soil’s skeleton and lowering of pore pressure to the zero level (when samples were taken from below groundwater level).
7. A method was proposed to calculate the reinforcement from FRP soil’s settlement. The determination of the soil–FRP preliminary reinforcement bearing capacity was undertaken in the initial study by Vatin and Nurmukhametov [37] and could be developed further. However, a calculation method for the bearing capacity has not been established and needs to be developed.

## Figures and Tables

**Table 1:** Summary of the investigated samples.

Sample Number	Short Name	Description	Reinforcement Factor, µ
1	Test wo reinf µ = 0	Reference sample without reinforcement	0.00
2	Test1 reinf µ = 0.22	Sample with one reinforcing element, length 25 cm	0.22
3	Test3 reinf µ = 0.69	Sample with three reinforcing elements, length 25 cm	0.69
4	Test9 reinf µ = 0.92	Sample with nine reinforcing elements, length 25 cm	0.92
5	Test27 reinf µ = 1.20	Sample with 27 reinforcing elements, length 25 cm	1.20
6	Test27 reinf edge40 cm µ = 1.39	Sample with 15 reinforcing elements (length 25 cm) and 12 elements at the edge (length 40 cm)	1.39
7	Test27 reinf mid40 cm µ = 1.43	Sample with 15 reinforcing elements (length 25 cm) and 12 elements in the middle (length 40 cm)	1.43

**Table 2:** Summary of the reference soil’s physical-mechanical properties.

Index	Name	W	ρ, t/m^3^	e	E, MPa
mL IV	Fine-grained sand, middle compaction	0.241	1.55	0.795	7.8

**Table 3:** Summary of the deformation modulus *E* from test results at a loading pressure of 100–200 kPa.

Reinforcement factor, µ	0	0.22	0.69	0.92	1.20	1.39	1.43
Deformation modulus E, MPa	0.87	1.01	1.42	1.66	3.37	5.27	4.39

## Data Availability

All data are available in the paper.
